# Prevalence of autism spectrum disorder in mainland china over the past 6 years: a systematic review and meta-analysis

**DOI:** 10.1186/s12888-024-05729-9

**Published:** 2024-05-29

**Authors:** Xinhong Jiang, Xianrui Chen, Jingying Su, Nan Liu

**Affiliations:** 1 Department of Rehabilitation, Fujian Medical University Union Hospital, Fuzhou, 350001 Fujian China; 2 Department of Pediatric Rehabilitation, Xiamen Rehabilitation Hospital, Xiamen, 361003 Fujian China; 3 Department of Pediatrics, The First Affiliated Hospital of Xiamen University, Xiamen, 361003 Fujian China; 4 Fujian Institute of Cerebrovascular Disease, Fuzhou, 350001 Fujian China; 5 Key Laboratory of Brain Aging and Neurodegenerative Diseases, Fujian Key Laboratory of Molecular Neurology, Fujian Medical University, Fuzhou, 350001 Fujian China; 6 Department of Neurology, Fujian Medical University Union Hospital, Fuzhou, 350001 Fujian China

**Keywords:** Autism spectrum disorder, Autism, Children, Prevalence, Systematic review, Meta-analysis

## Abstract

**Background:**

Coupled with its rising prevalence, Autism spectrum disorder (ASD) has become a globally recognized public health concern. Nevertheless, large-scale, multicenter studies that analyze the epidemiology of ASD in China are relatively scarce.

**Methods:**

Literature searches were conducted in PubMed/Medline, Embase, the Cochrane Library, Wanfang Data Knowledge Service Platform, China Biology Medicine database (CBM), China Science and Technology Journal Database (CSTJ), and China National Knowledge Infrastructure (CNKI) to retrieve studies published before April 8, 2023, related to ASD prevalence among children aged 0 to 14 years in mainland China. Meta-analysis was conducted using RevMan 5.2 and Stata 14.0.

**Results:**

Twenty-one articles were included. The ASD prevalence among children in mainland China has been 0.7% (95% confidence interval(CI): 0.006–0.008) since 2017. The prevalence of ASD among boys was 1.0% (95% CI: 0.008–0.011), which was significantly higher than that among girls at 0.2% (95% CI: 0.002–0.003), with a statistically significant difference (OR = 3.198, 95% CI: 2.489–4.109, *P* = 0.000). Among the included studies, 18 reported an ASD prevalence of 0.8% (95% CI: 0.007–0.010), while 3 studies reported an autistic disorder (AD) prevalence of 0.7% (95% CI: 0.006–0.008). The prevalence of autism among urban children was 23.9% (95% CI: 0.149–0.328), and in rural areas, it was 0.7% (95% CI: 0.002–0.013), with no statistically significant difference (OR = 1.342, 95% CI: 0.258–6.975, *P* = 0.727). Regression analysis showed that factors such as region (*P* = 0.000), age (*P* = 0.000), study period (*P* = 0.000), sample size (*P* = 0.000), sampling method (*P* = 0.002), population source (*P* = 0.000), disease type (*P* = 0.000), quality score of the study (*P* = 0.000), and diagnostic criteria (*P* = 0.000) might have contributed to the heterogeneity in ASD prevalence.

**Conclusion:**

The prevalence of ASD in China from 2017 to 2023 was 7/1000, showing an upward trend compared to that before 2017 (26.50/10,000). The male-to-female prevalence ratio was 5:1.The overall prevalence remained significantly lower than that reported in foreign countries.

**Supplementary Information:**

The online version contains supplementary material available at 10.1186/s12888-024-05729-9.

## Background

Autism spectrum disorder (ASD) is a widespread neurodevelopmental disorder that primarily occurs in early childhood. It is also referred to as autism or autistic disorder. The main characteristics of ASD include difficulties in speech communication, impaired social interactions, restricted interests, repetitive behaviors, and often intellectual challenges. Subtypes of ASD include autistic disorder (AD), Asperger syndrome, and pervasive developmental disorder not otherwise specified (PDD-NOS) [1, 2]. Increasing evidence suggests that ASD frequently coexists with symptoms or conditions that cannot be solely explained by ASD itself, known as comorbidities. These comorbidities include attention-deficit hyperactivity disorder [3, 4], epilepsy [5, 6], sleep disorders [7, 8], and gastrointestinal issues [9–11]. Children with ASD often experience one or more coexisting conditions, which may vary over time and mutually influence each other, indicating a poorer prognosis for affected individuals [12].

ASD typically manifests before the age of 3 and substantially impacts the physical and mental health of children and adolescents. Due to its poor prognosis, high disability rate, and the need for long-term rehabilitation, ASD imposes considerable economic and psychological burdens on both society and families. Coupled with its rising prevalence, ASD has become a globally recognized public health concern [13]. Reported prevalence rates of ASD vary across different countries and regions. The global prevalence is estimated to be approximately 1% [14]. A study by Liu Xian et al. [15] reported an ASD prevalence of 26.50/10,000 in China from 2000 to December 2016. In 2023, the Autism and Developmental Disorders Monitoring Network (ADDM) in the United States released data for 11 states in 2020, showing an overall ASD prevalence of 27.6/1000 aged 0–8 years (1/36) [16]. This marked an increase of 17.8% from the 2018 prevalence of 22.7/1000 (1/44) [17] and a 32.97% increase from the 2016 prevalence [18] of 18.5/1000 (1/54).

Currently, reports on ASD prevalence or screening rates vary in different regions within China. For instance, Zheng Kai et al. [19] reported an ASD prevalence of 1.15/1000 in the Ningbo region, while Deng Cheng et al. [20] found a positive ASD screening rate of 10.2/1000 preschool children in Zhongshan city. Nevertheless, large-scale, multicenter studies that analyze the epidemiology of ASD in China are relatively scarce. This study aimed to comprehensively analyze the relevant literature through quantitative methods, exploring the recent prevalence of ASD among children in mainland China. The goal is to provide evidence-based data support for assessing health care service demands and developing interventions, treatments, and further research endeavors.

## Methods

### Literature search strategy

#### Computerized database search

We conducted searches in databases including PubMed/Medline, Embase, the Cochrane Library, Wanfang Data Knowledge Service Platform, China Biology Medicine database (CBM), China Science and Technology Journal Database (CSTJ), and China National Knowledge Infrastructure (CNKI). Additionally, we traced the references of the included studies. The search period was limited to records published up to May 8, 2023. The search strategy involved a combination of subject terms and free-text keywords. Furthermore, relevant references from the included studies were manually searched.

The English search terms used were “Autistic Disorder,” “Autism,” “Disorder, Autistic,” “Disorders, Autistic,” “Kanner’s Syndrome,” “Kanner Syndrome,” “Autism, Infantile,” “Infantile Autism,” “Autism, Early Infantile,” “Early Infantile Autism,” “Infantile Autism, Early,” “autism spectrum disorder,” “Asperger,” “Asperger syndrome,” “childhood disintegrative disorder,” “pervasive developmental disorder not otherwise specified,” “Rett syndrome,” “PDD-NOS,” “China,” and “Chinese.”

Using PubMed/Medline as an example, the search query was as follows:

(“Autistic Disorder“[Mesh]) OR “Autism“[All Fields] OR “Disorder, Autistic“[All Fields] OR “Disorders, Autistic“[All Fields] OR “Kanner’s Syndrome“[All Fields] OR “Kanner Syndrome“[All Fields] OR “Autism, Infantile“[All Fields] OR “Infantile Autism“[All Fields] OR “Autism, Early Infantile“[All Fields] OR “Early Infantile Autism“[All Fields] OR “Infantile Autism, Early“[All Fields] OR “autism spectrum disorder“[All Fields] OR “Asperger“[All Fields] OR “Asperger syndrome“[All Fields] OR “childhood disintegrative disorder“[All Fields] OR “pervasive developmental disorder not otherwise specified“[All Fields] OR “Rett syndrome“[All Fields] OR “PDD-NOS“[All Fields]) AND (“prevalence“[All Fields] OR “morbidity“[All Fields] OR “epidemiology“[All Fields] OR “cross-sectional study“[All Fields]) AND (“china“[All Fields] OR “chinese“[All Fields]).

The inclusion criteria for the studies was as follows: (1) studies that clearly reported the prevalence of ASD or its subtypes; (2) study participants who were children aged < 14 years from mainland China; (3) studies published in either Chinese or English; and (4) full-text articles published between January 1, 2017, and April 8, 2023.

The exclusion criteria for the studies was as follows: (1) studies with incomplete data; (2) duplicate studies or data, such as repeated data from the same study or retaining only the study with the largest sample size for the same population; and (3) secondary sources such as reviews, case reports, and conference proceedings.

### Literature screening, data extraction, and quality assessment

Two clinical physicians independently performed the literature screening, data extraction, and quality assessment. A third clinical physician verified the process. In case of any discrepancies, they jointly discussed and resolved the differences, or a third physician analyzed and made the final judgment. The initial screening involved excluding obviously irrelevant articles based on their titles and abstracts. Subsequently, the remaining articles were further reviewed by reading their full texts.

The extracted information from the included articles comprised the following aspects: general information, including the first author, publication date, study period, region (east/west/south/north/central/mixed), sample size, sex, age, etc., and the outcome measurement of the prevalence of autism among children.

The quality assessment of the included articles was conducted according to the criteria from the Agency for Healthcare Research and Quality (AHRQ) [21, 22]. This assessment consists of 11 items, each scored as 2 points for “yes” (meeting the criteria), 0 points for “no” (not meeting the criteria), and 1 point for “unclear” (not described in the article). The total score ranges from 0 to 22 points. Articles that scored 0–7 points were considered low quality, 8–14 points were considered moderate quality, and 15–22 points were considered high quality. This assessment was independently conducted by two evaluators. In case of discrepancies, they discussed with the third researcher until a consensus was reached.

### Statistical analysis

Statistical data analysis was conducted using Review Manager 5.2 and Stata 14.0. For categorical data, odds ratios (OR) were used, and for continuous data, standardized mean differences (SMD) were employed. Effect sizes are presented with point estimates and their corresponding 95% confidence intervals (CI). Heterogeneity analysis was performed using the Q test and I^2^ test. If I^2^ = < 50%, indicating no statistically significant heterogeneity, a fixed-effects model was used. If I^2^ > 50%, indicating significant heterogeneity, a random-effects model was employed. Subgroup analysis or sensitivity analysis was conducted to explore the reasons for heterogeneity. Publication bias was assessed using funnel plots or Egger’s test. A significance level of *P* < 0.05 was considered statistically significant for differences.

## Results

### Literature search results and quality assessment

**Fig. 1:**
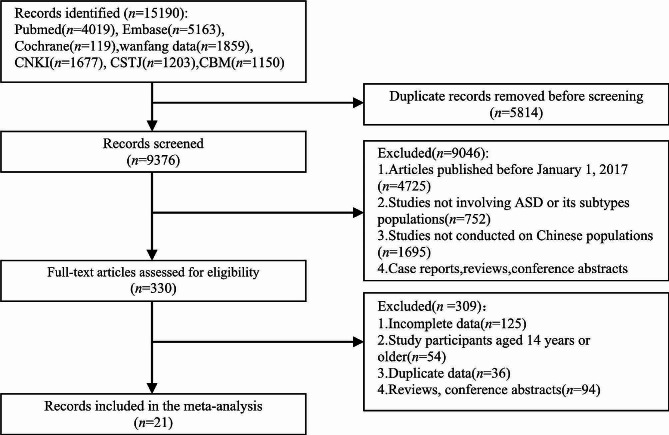
Flow chart of literature screening

The initial search yielded a total of 15,190 relevant articles, of which 330 articles underwent full-text screening. Eventually, 21 articles met the inclusion criteria and were included in this systematic review. These studies encompassed a total of 349,900 children from mainland China. The quality assessment scores of the included articles ranged from 11 to 19 points, with the majority falling within the range of 12 to 17 points. The article selection process is depicted in Fig. 1. The basic information of the included articles and their quality assessment results are presented in Table 1.

**Table 1:** Information and quality of studies included for the systematic review

First Author	Publication Year	Provinces	Region	Age (years)	Sample source	Sample selection method	AS/ASD	Screening/ diagnostic tools	Diagnostic criteria	Quality score		total			Male			Famale	
												sample size	event		sample	event		sample	event
Chen GH [23]	2022	Jining	Northern	3∼6	K/S	CS	ASD	CABS、CARS、ABC	ICD-10	16		14,263	94		7788	82		6475	12
Zhang YQ [24]	2021	Dongguan	Eastern	0.5∼3	H	NA	ASD	M-CHAT、ABC、CARS	DSM-V	16		2000	27		1120	21		880	6
Hao JQ [25]	2021	Inner Mongolia	Northern	3∼14	K/S	RCS	ASD	CABS、ABC、CARS	DSM-5、 DSM-IV	16		7108	26		3859	17		3249	9
Ding N [26]	2021	Wuhu	Midst	2∼6	H	NA	ASD	CARS	DSM-5	14		12,657	52		6974	47		5683	5
Zou Y [27]	2021	Panzhihua	Western	1.5∼3	C	SCS	ASD	M-CHAT、ABC、CARS、SPT、GDS	DSM-5	14		1957	11		1112	8		845	3
Cao CH [28]	2021	Xi’an	Northern	3∼7	K/S	SCS	ASD	ABC、ADOS-2	DSM-V	19		5178	38		2682	33		2496	5
Ying YJ [29]	2021	Yongkang	Eastern	1.5∼3	C	NA	Autism	M-CHAT、ABC、SCQ、	DSM-5	14		15,484	46		8067	35		7417	11
Yuan LH [30]	2021	Huizhou	Eastern	4∼6	K/S	RCS	ASD	CABS、CARS、	DSM-IV	12		7241	380		3960	252		3281	128
Long SM [31]	2021	Kaili	Southern	1.5∼ 2.5	H	NA	ASD	WSCMBD、M-CHAT、ABC	NA	11		6000	39		3622	28		2378	11
Liu YM [32]	2021	Lanzhou	Northern	6∼11	C	CS	ASD	CAST	CCMD-3	14		2486	23		1302	15		1184	8
Zhou H [33]	2020	five province and three municipalities	mixed	6∼12	C + K/S	SCS	ASD	MC-ASRS、ADOS、ADI-R	DSM-V	19		125,806	363		66,687	292		59,119	71
Shen JH [34]	2020	Qingyuan	Eastern	2∼6	K/S + H	NA	ASD	ABC	DSM-5	15		4512	49		2564	41		1948	8
Liu D [35]	2019	Shaoxing	Eastern	1.3∼2.5	H	NA	ASD	CARS、M-CHAT-R/F	DSM-5	15		1944	5		1062	4		882	1
A HH [36]	2019	Qinhai	Northern	0∼6	C	RS	Autism	CARS、ABC	ICD-10	14		3944	5		2033	4		1911	1
Liang LD [37]	2019	Wenzhou	Eastern	6∼12	C	RCS	ASD	WISC-R、ASRS、ADOS、ADI-R	DSM-V	16		13,987	25		7006	21		6981	4
Jin Z [38]	2018	Shanghai	Eastern	3∼12	K/S	SCS	ASD	SCQ、Gesell test、WPPSI、WISC-R、	DSM-5	19		74,252	203		39,038	157		35,241	46
Sun P [39]	2018	Yongchuan	Western	2∼6	C	SCS	ASD	CABS	CARS	14		7500	56		-	37		-	19
Yang CJ [40]	2018	Chongqing	Western	2.5∼6	K/S	SCS	ASD	SRS、SCQ、ABC、CARS	DSM-5	17		6212	22		3308	17		2912	5
Wen HY [41]	2018	Beijing	Northern	2∼6	K/S	RCS	Autism	M-CHAT、CABS、ABC	DSM-5	17		11,770	33		6124	24		5646	9
Geng XM [42]	2018	Zhuhai	Eastern	1.5∼3	C	RSS	ASD	M-CHAT、ABC、CARS、	CARS	12		2000	97		1235	82		765	15
Li L [43]	2017	Hainan	Eastern	0∼6	C	SCS	ASD	WSCMBD	DSM-V	16		37,862	235		20,824	206		17,038	29

Sample source: C, community-based; K/S, kindergarten/School; H, hospital physcial examination clinic. Sample selection method: SCS, Stratified cluster sampling; RCS, Random cluster sampling; RS, Random sampling; RSS, Random stratified sampling; CS, Cluster sampling; NA, Not available. Screening/diagnostic tools: ABC, Autism Behavior Checklist; CABS, Clancy Autism Behavior Scale; CARS, Childhood Autism Rating Scale; CHAT, Checklist for Autism in Toddlers; M-CHAT, Modified Checklist for Autism in Toddlers; CAST, Children Autism Spectrum Test; ASSQ, high function Autism Spectrum screening questionnaire; SCQ, Social Communication Questionnaire; SPT, symbolic play test; GDS, Gesell Developmental Schedules; ADOS-2, autism diagnostic observation schedule,2nd edition; ADI-R, Autism Diagnostic Interview-Revised; ASRS, Social Behavior and Communication Skills Screening Questionnair; WlSC-R, Wechsler Intelligence Scale for Children; SRS, Social Responsiveness Scale; WPPSI, Wechsler Preschool and Primary Scale of Intelligence; WSCMBD, warning sign for children mental and behavioral development Screening Questionnaire. Diagnostic criteria: CCMD-3, Chinese Classification of Mental Disorders, 3rd edition; DSM-III-R, Diagnostic and Statistical Manual of Mental Disorders, 3rd edition, revised; DSM-IV, Diagnostic and Statistical Manual of Mental Disorders, 4th edition; DSM-V, Diagnostic and Statistical Manual of Mental Disorders, 5th edition; ICD-10, International Classification of Diseases, 10th revision.

## Meta-analysis results

In this study, meta-analyses were conducted for the prevalence of autism among children, the prevalence of autism among children of both sexes, and the prevalence of autism among urban and rural children. The results were as follows:

### Meta-analysis results of childhood autism prevalence

**Fig. 2:**
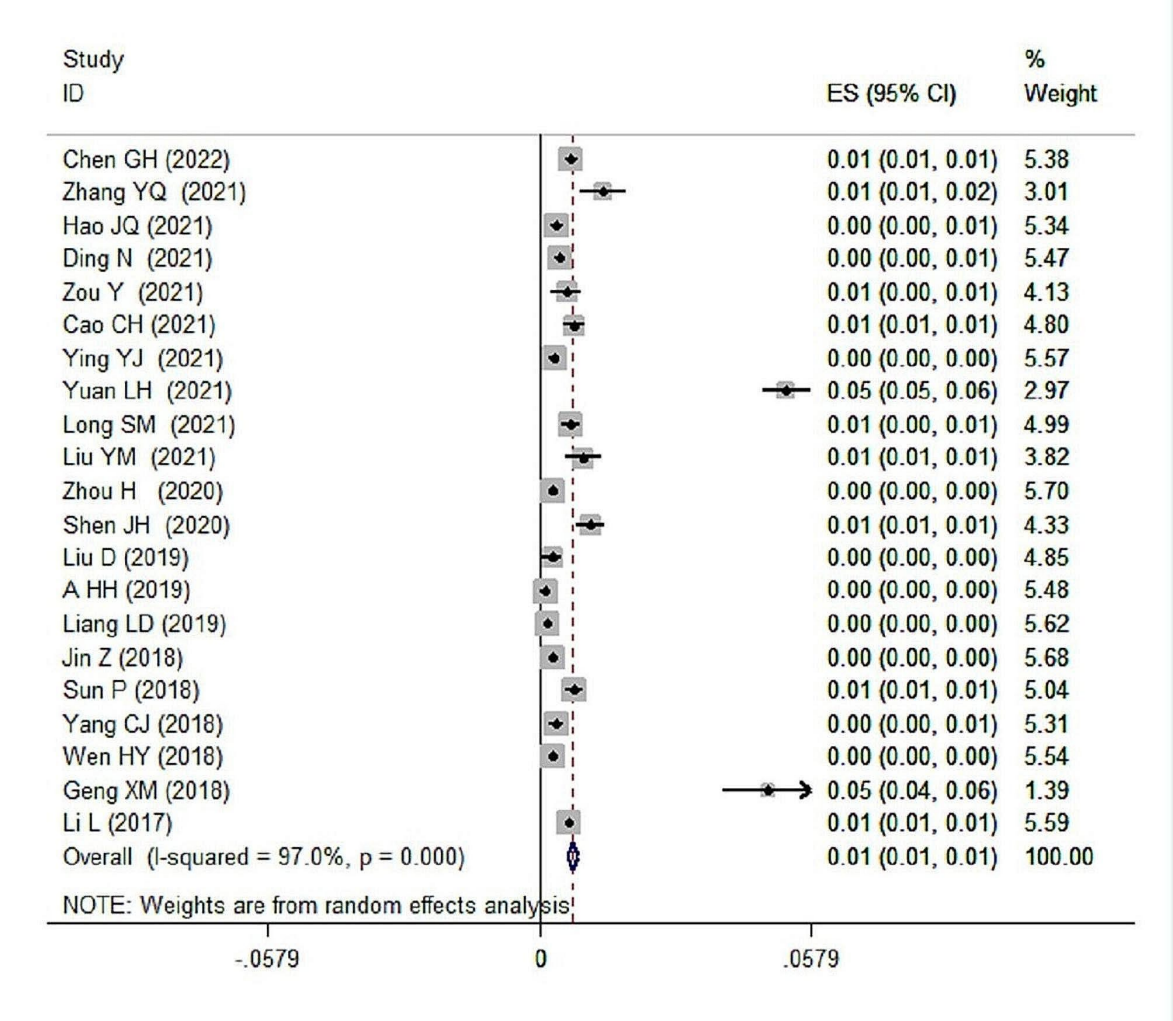
Meta-analysis forest plot of the prevalence of childhood autism spectrum disorder (ASD) in mainland China

Heterogeneity test was conducted on the 21 included articles, and the results showed an I^2^ of 97.0% with a p value of 0.000, indicating significant heterogeneity. Sensitivity analysis was performed on the included articles, and the results remained consistent, confirming the utilization of a random-effects model for meta-analysis. The meta-analysis results revealed a prevalence rate of 0.7% (95% CI: 0.006–0.008) for childhood autism in China (Fig. 2). Sensitivity analysis demonstrated that the exclusion of any single article had no impact on the combined effect size.

Subgroup analyses were conducted based on factors including region(Eastern/Western/Southern/Northern/Midst/mixed), age (0–3 years/4–6 years/7–14 years/mixed ages), sample source (community-based/kindergarten or school/hospital physical examination clinic/mixed sources), sample selection method (stratified cluster sampling(SCS)/random cluster sampling(RCS)/ random sampling(RS)/random stratified sampling(RSS)/cluster sampling(CS)/not available(NA)), disease type (ASD/autism), diagnostic criteria (DSM-IV/DSM-V/ICD-10/CCMD-3/other), quality score ( > = 15 points/<15 points), and sample size (< 5000/>=5000–10,000/>=10,000). All subgroup analyses exhibited significant heterogeneity; thus, the random-effects model was employed to combine effect sizes (as shown in Table 1).

### Region analysis

**Fig. 3:**
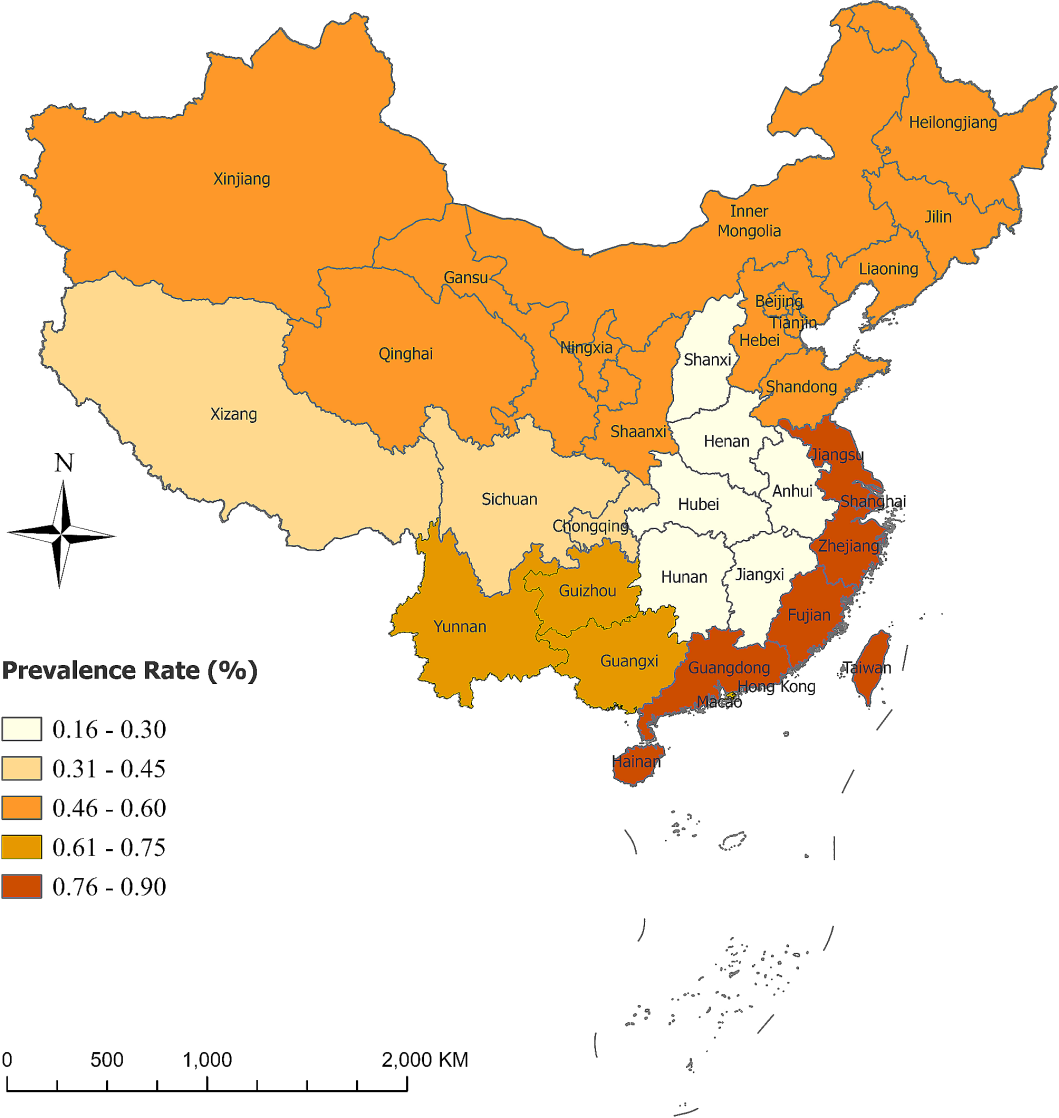
Prevalence map of childhood autism spectrum disorder (ASD) in various regions in mainland China

The eastern region had a prevalence rate of 0.9% (95% CI: 0.007–0.011) from 10 studies. The western region had a prevalence rate of 0.4% (95% CI: 0.002–0.005) from 2 studies. The southern region had a prevalence rate of 0.7% (95% CI: 0.004–0.009) from 1 study. The northern region had a prevalence rate of 0.5% (95% CI: 0.003–0.006) from 7 studies. The midst region had a prevalence rate of 0.3% (95% CI: 0.002–0.005) from 2 study. (Refer to Table 1; Fig. 3)

### Age analysis

The 0–3 years had a prevalence rate of 1.1% (95% CI: 0.006–0.015) from 6 studies. The 4–6 years had a prevalence rate of 2.2% (95% CI: 0.005–0.039) from 3 studies. The 7–14 years had a prevalence rate of 0.3% (95% CI: 0.002–0.005) from 3 studies. Mixed age had a prevalence rate of 0.4% (95% CI: 0.003–0.006) from 9 studies.

### Sample source analysis

Community-based sources showed a prevalence rate of 0.7% (95% CI: 0.004–0.009) from 8 studies. Kindergartens showed a prevalence rate of 0.9% (95% CI: 0.006–0.012) from 8 studies. Hospital physical examination clinics showed a prevalence rate of 0.7% (95% CI: 0.002–0.012) from 3 studies. Mixed sources showed a prevalence rate of 0.7% (95% CI: -0.001-0.015) from 2 studies.

### Sampling selection method analysis

Stratified cluster sampling showed a prevalence rate of 0.5% (95% CI: 0.004–0.006) from 7 studies. Random cluster sampling showed a prevalence rate of 1.4% (95% CI: 0.007–0.021) from 4 studies. Cluster sampling showed a prevalence rate of 4.9% (95% CI: 0.039–0.058) from 1 study. The prevalence rates of other methods are shown in Table 1. Unclear sampling methods showed a prevalence rate of 0.6% (95% CI: 0.004–0.008).

### Disease type analysis

ASD had a prevalence rate of 0.8% (95% CI: 0.007–0.010) from 18 studies. Autism had a prevalence rate of 0.7% (95% CI: 0.006–0.008) from 3 studies.

### Diagnostic criteria analysis

The DSM-IV showed a prevalence rate of 5.2% (95% CI: 0.047–0.058) from 1 study. The DSM-V showed a prevalence rate of 0.4% (95% CI: 0.003–0.005) from 14 studies. The ICD-10 showed a prevalence rate of 0.4% (95% CI: -0.001-0.009) from 2 studies. The CCMD-3 showed a prevalence rate of 0.9% (95% CI: 0.005–0.013) from 1 study. Other criteria showed a prevalence rate of 1.8% (95% CI: 0.009–0.028) from 3 studies.

### Quality score analysis

Studies with a quality score > = 15 points showed a prevalence rate of 0.5% (95% CI: 0.004–0.005) from 12 studies. Studies with a quality score < 15 points showed a prevalence rate of 1.4% (95% CI: 0.009–0.018) from 9 studies.

### Sample size analysis

Studies with a sample size < 5000 showed a prevalence rate of 1.1% (95% CI: 0.006–0.017) from 7 studies. Studies with a sample size > = 5000–10,000 showed a prevalence rate of 1.3% (95% CI: 0.006–0.020) from 6 studies. Studies with a sample size > = 10,000 showed a prevalence rate of 0.4% (95% CI: 0.003–0.005) from 8 studies (Table 2).Further meta-regression analysis was conducted, and the results indicated that factors such as region (*P* = 0.000), age (*P* = 0.000), publication year (*P* = 0.000), sample size (*P* = 0.000), sampling selection method (*P* = 0.002), sample source (*P* = 0.000), disease type (*P* = 0.000), quality score (*P* = 0.000), and diagnostic criteria (*P* = 0.000) contributed to the heterogeneity in the prevalence of childhood ASD rates (Table 3).

**Table 2:** Summary table of subgroup analysis of the prevalence of autism in children in mainland China

Subgroup factors	Studies Included	Sample size	Event	Heterogeneity test			Meta-analysis		Effect model
				I^2^(%)	P value		Prevalence(%)	95%CI	
Region									
Eastern	9	159,282	1067	98.6	0.000		1.2	0.009–0.015	Random
Western	3	15,669	89	80.1	0.000		0.5	0.003–0.008	Random
Southern	1	6000	39	-	-		0.7	0.004–0.009	Random
Northern	6	44,749	219	91.3	0.000		0.5	0.003–0.007	Random
Midst	1	12,657	52	-	-		0.4	0.003–0.005	Random
mixed	1	125,806	363	-	-		0.3	0.003–0.003	Random
**Age**									
0–3	6	29,385	225	95.6	0.000		1.1	0.006–0.015	Random
4–6	3	26,682	512	99.3	0.000		2.2	0.005–0.039	Random
6–14	3	142,279	411	89.8	0.000		0.3	0.002–0.005	Random
mixed	9	165,817	681	93.2	0.000		0.4	0.003–0.006	Random
**Sample source**									
C	8	85,220	498	96.5	0.000		0.7	0.004–0.009	Random
K/S	8	138,681	848	98.2	0.000		0.9	0.006–0.012	Random
H	3	9944	71	88.3	0.000		0.7	0.002–0.012	Random
mixed	2	130,318	412	96.2	0.000		0.7	-0.001-0.015	Random
**Sample selection method**									
SCS	7	258,767	928	93.9	0.000		0.5	0.004–0.006	Random
RCS	4	40,106	464	99.2	0.000		1.4	0.007–0.021	Random
RS	1	3944	5	-	-		0.1	0.000-0.002	Random
RSS	1	2000	97	-	-		4.9	0.039–0.058	Random
CS	2	16,749	117	41.5	0.000		0.7	0.005–0.010	Random
NA	6	42,597	218	89.2	0.000		0.6	0.004–0.008	Random
**Diseases type**									
ASD	18	332,965	1745	97.4	0.000		0.8	0.007–0.010	Random
Austim	3	31,198	84	68.1	0.000		0.7	0.006–0.008	Random
**Diagnostic criteria**									
1 DSM-IV	1	7241	380	-	-		5.2	0.047–0.058	Random
2 DSM-V	14	320,729	1135	90.7	0.000		0.4	0.003–0.005	Random
3 ICD-10	2	18,207	99	97.2	0.000		0.4	-0.001-0.009	Random
4 CCMD-3	1	2486	23	-	-		0.9	0.005–0.013	Random
0 other	3	15,500	192	97.3	0.000		1.8	0.009–0.028	Random
**Quality score**									
>=15	12	304,894	1120	93.1	0.000		0.5	0.004–0.005	Random
< 15	9	59,269	709	98.3	0.000		1.4	0.009–0.018	Random
**Sample size**									
< 5000	7	18,843	217	96.1	0.000		1.1	0.006–0.017	Random
>=5000–10,000	6	39,239	561	98.5	0.000		1.3	0.006–0.020	Random
>=10,000	8	306,081	1051	93.6	0.000		0.4	0.003–0.005	Random

**Table 3:** Results of regression analysis of the prevalence of autism in children in mainland China

Covariate	Meta-regression coefficient	95%Confidence	P value
		interval	
Region	-4.990	0.00076 to 0.05586	0.000
Age	-11.370	0.00127 to 0.01024	0.000
Sample source	-7.390	0.00126 to 0.02449	0.000
Sample selection method	-3.630	0.00821 to 0.28653	0.002
Diseases	-11.100	0.00076 to 0.00741	0.000
Diagnostic criteria	-6.530	-0.00202 to -0.04230	0.000
Quality score	-15.740	0.00450 to 0.01599	0.000
Sample size	-13.940	0.00389 to 0.01665	0.000

### Analysis results of the comparison of the prevalence of ASD among children of both sexes

**Fig. 4:**
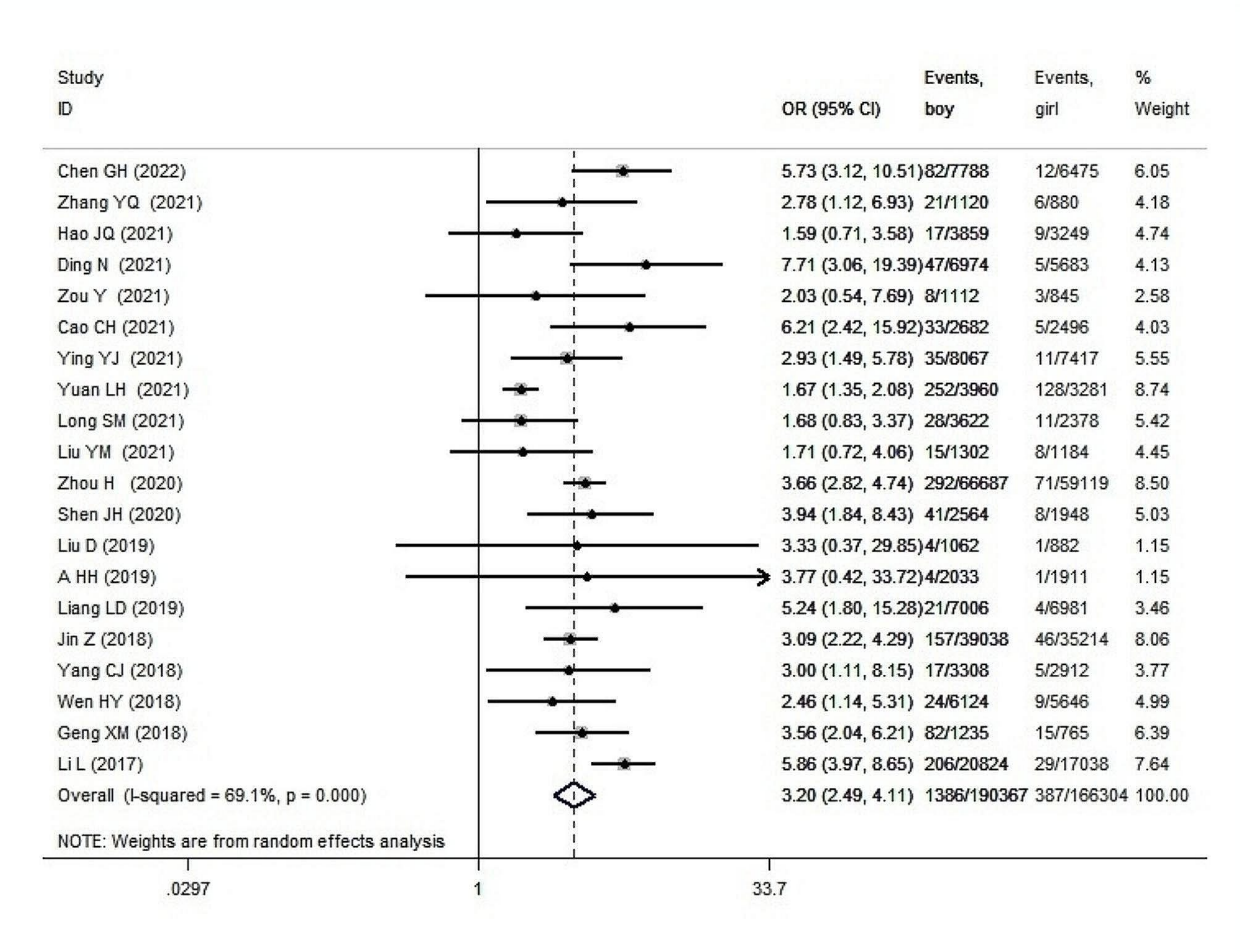
Meta-analysis forest plot of the prevalence of autism in children of different genders in mainland China

A total of 20 studies reported the prevalence rates of ASD among boys and girls. The heterogeneity test yielded a P value of 0.000 and an I² value of 69.1%, indicating significant heterogeneity. A random-effects model was employed for the analysis. The meta-analysis results revealed that the prevalence of ASD among boys was significantly higher than that among girls (OR = 3.198, 95% CI: 2.489 to 4.109, *P* = 0.000) (Fig. 4). Sensitivity analysis indicated that excluding any single study did not significantly affect the combined effect value.

Subgroup analysis were conducted based on region (Eastern/Western/Southern/Northern/Midst/mixed), age (0–3 years/4–6 years/7–14 years/mixed ages), sample source (community-based/kindergarten or school/hospital physical examination clinic/mixed), sampling selection method (SCS/RCS/RS/RSS/CS/NA), disease type (ASD/autism), and diagnostic criteria (DSM-IV/DSM-V/ICD-10/CCMD-3/other). The results of subgroup analyses showed significant heterogeneity; therefore, a random-effects model was used to combine effect sizes. The subgroup analysis results are presented in Supplement 1.

Further meta-regression analysis was conducted, and the results indicated that region (*P* = 0.007), age (*P* = 0.002), sampling selection method (*P* = 0.009), and disease type (*P* = 0.010) contributed to the heterogeneity in childhood autism prevalence rates (Supplement 1).

### Analysis results of the comparison of the prevalence of ASD between urban and rural children

**Fig. 5:**
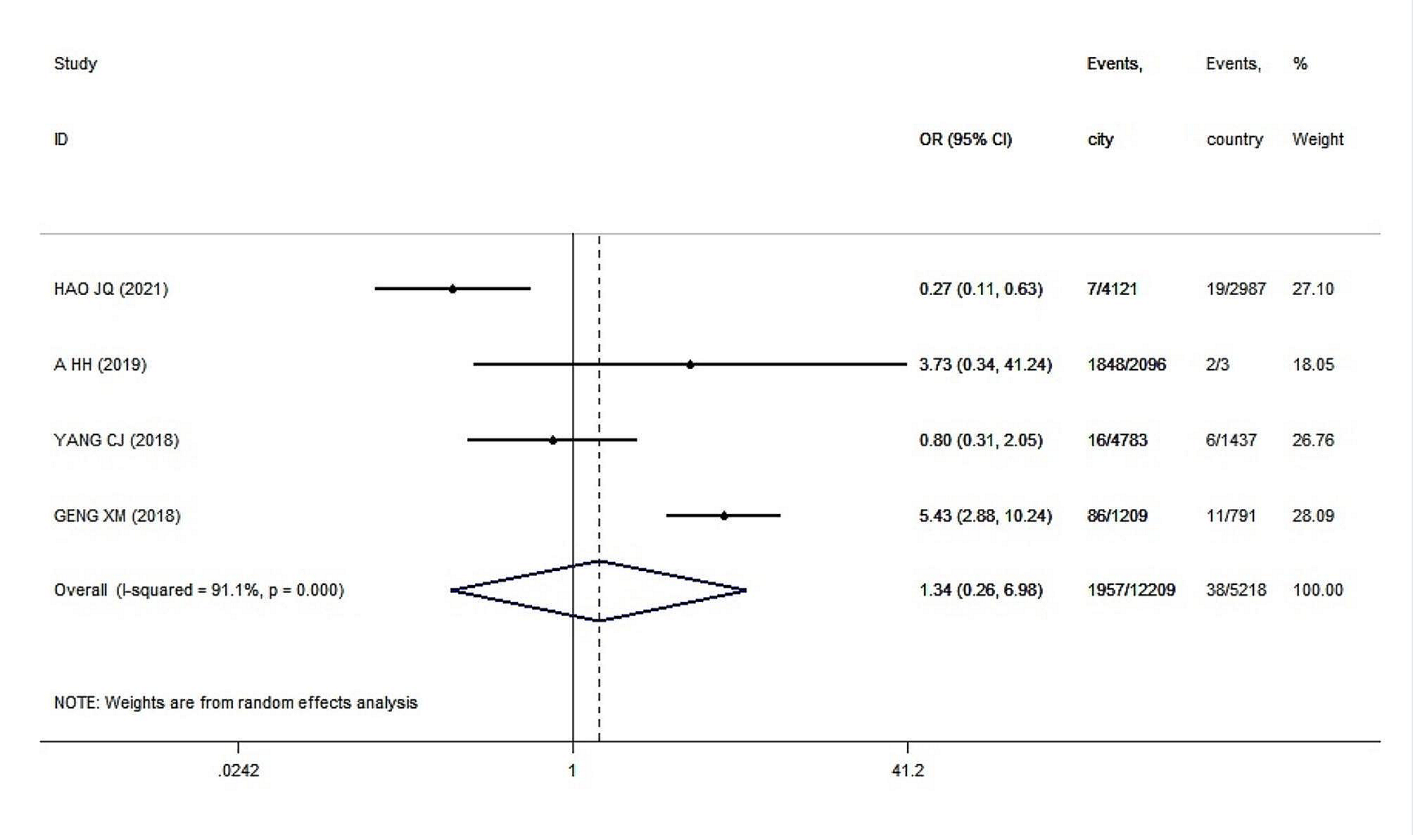
Meta-analysis forest plot of the prevalence comparison of autism between urban and rural children in mainland China

Four studies reported the prevalence of ASD among urban and rural children. The test for heterogeneity showed *P* = 0.000 and I^2^ = 91.9%, indicating significant heterogeneity. The random-effects model was used for the meta-analysis. The results of the meta-analysis showed that there was no statistically significant difference in the prevalence of ASD between urban and rural children (OR = 1.342, 95% CI: 0.258–6.975, *P* = 0.727), as depicted in Fig. 5. Sensitivity analysis indicated that the exclusion of any single study did not significantly affect the combined effect size.

### Publication Bias

**Fig. 6:**
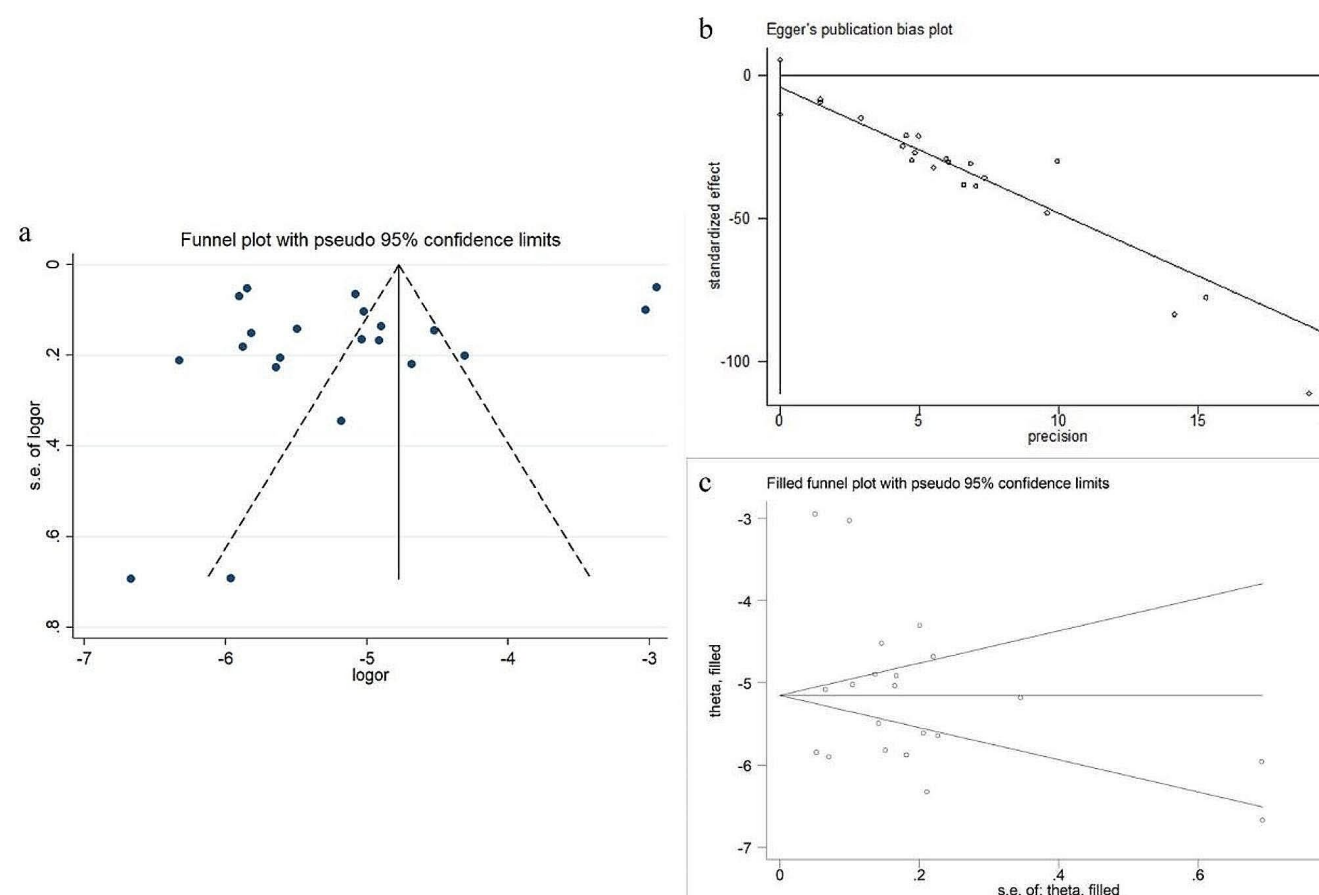
Publication bias test for the prevalence of autism in children. (a) Funnel Plot. (b) Egger Plot. (c) Trim and Fill Method

A funnel plot test was conducted to assess publication bias in the meta-analysis of the overall prevalence of childhood ASD. The funnel plot (Fig. 6a) displayed an asymmetric distribution, suggesting the possibility of publication bias. Further analysis using Egger’s test showed a t value of -0.96 and a P value of 0.349, indicating that the difference in publication bias was not statistically significant (Fig. 6b). The results of the trim-and-fill method also demonstrated that the combined effect size and P value remained relatively stable before and after adjustment (Fig. 6c), suggesting that the conclusions of this meta-analysis are relatively robust.

## Discussion

This study included a total of 21 articles [23–43] that included 349,900 children from 11 provinces, 3 municipalities directly under the central government, and 1 autonomous region in mainland China. Compared to previous meta-analyses [15, 44–46], this study evaluated the literature within the past 6 years, expanded the age range of inclusion compared to the study by Shi Huifeng et al. [45], and further subdivided potential subgroup factors that could affect the prevalence of ASD, such as age, diagnostic criteria, region, sample size, and quality scores. The results of this study showed that the prevalence of ASD among children was 5/1000 from 2017 to 2019 and 9/1000 from 2019 to the present. The overall prevalence of ASD over the past 6 years was 7/1000, which represents an upward trend compared to those in the study by Liu Xian et al. [15] in 2017 (2.65/1000) and the study by Hao Xiaohui et al. [44] in 2015 (2.88/1000). This trend is consistent with the gradually increasing global prevalence of ASD [14].

The increase in the prevalence of ASD among Chinese children in recent years may be attributed to several factors. First, the diagnostic criteria for ASD have been continuously revised, broadening the scope of the disorder. Second, the level of screening and diagnosis for ASD has improved. Third, the awareness of ASD among health care professionals and parents has increased, leading to better recognition and awareness. However, the prevalence of ASD among Chinese children remains lower than that in other countries worldwide. ADDM Network in the United States reported that the prevalence of ASD has increased annually, reaching 14.6/1000 (1/68) in 2012, 16.8/1000 (1/59) in 2014, 18.5/1000 (1/54) in 2016, 22.7/1000 (1/44) in 2018, and 27.6/1000 (1/36) in 2020 [16–18, 47, 48]. China’s current prevalence rate of ASD is still lower than the rate in the United States 11 years ago. The prevalence rate in the United Kingdom is also higher than that in China [49]. Studies by Uchan et al. [50] found an ASD prevalence rate of 6/1000 in North America and a median incidence rate of 6.19/1000 in Europe [51]. In Asia, South Korea reported a prevalence rate of 26.4/1000 children, which is also increasing [52, 53]. A study in Japan reported a prevalence rate of 32.2/1000 for ASD among children under 5 years [54]. Worldwide, the median male-to-female ratio of ASD prevalence is 4.2 [14]. In the United States, the reported prevalence of ASD is 3.8 times higher among boys than among girls [16], and in the UK, the sex ratio for typical autism in children is 4:1 [55]. This study showed that the 4–6 years had the highest prevalence of 22/1000, followed by the 0–3 years with 11/1000 and the years and mixed ages. This finding is consistent with the results of a nationwide survey in China, which found the highest risk in the 4–6 age group [56].China’s lower prevalence of ASD compared to that in most other countries could be attributed to several factors. First, it may be related to the age distribution of the surveyed population, with a greater focus on the 2–6 years in China, whereas international studies cover a broader age range. Second, more severe cases of ASD are diagnosed in China, such as classic and severe autism [57], while milder forms such as Asperger syndrome and mild autism tend to be underdiagnosed, potentially leading to an underestimation of ASD prevalence. Third, most epidemiological surveys in China are conducted on a regional basis, lacking a nationwide survey system with reliable prevalence monitoring. Furthermore, China lacks standardized, reliable, and validated screening tools, whereas developed countries have comprehensive case monitoring systems and utilize different monitoring methods and survey techniques. Last, the awareness of ASD and autism among caregivers and health care professionals in China is still lower than that in developed countries, and the stigma associated with these conditions might lead to deliberate concealment of the condition by parents.

This study revealed that the prevalence of ASD in the eastern region of China was 12/1000, which was higher than that in the southern, northern, midst, and western regions. The observed disparities might be attributed to variations in awareness and diagnostic capabilities for ASD across different regions. However, the differences could also be linked to the relatively limited inclusion of ASD cases and sample sizes. Further research with larger sample sizes and additional subgroup analyses is needed to confirm the variations in ASD prevalence among different regions.

ASD screening tools are widely used in global epidemiological surveys, and the choice of different screening tools can substantially influence estimates of ASD prevalence [58]. CABS is the most commonly used ASD screening tool in China [59], but its sensitivity is relatively low, and it is rarely used in foreign studies. In contrast, tools such as ADOS and ADI-R provide more comprehensive information and are extensively used in foreign research [60]. Therefore, differences in the choice of ASD and autism screening tools partially explain the disparities between domestic and foreign study results [61]. The study by Liu Xian et al. [15] demonstrated that there was no statistically significant difference in prevalence rates when applying CCMD or DSM diagnostic criteria. In contrast, this meta-analysis showed that the prevalence of ASD using DSM-V diagnostic criteria was 4 per 1000 children, which was the same as that using the ICD-10 criteria but lower than that using the DSM-IV (based on only one study), CCMD-3 (based on only one study), and other diagnostic criteria. This suggests that different diagnostic criteria may lead to variations in prevalence rates.

Subgroup analysis based on sampling sources in this study indicated that the prevalence rates among kindergarten populations were higher compared to community-based, hospital physical examination clinic, and mixed populations. This contradicts the findings of previous meta-analyses [15, 46] that indicated higher prevalence rates among populations from hospitals. This discrepancy may be attributed to several factors: (1) a larger number of kindergarten-based studies were included in this research, predominantly focusing on preschool children aged 4–6 years; (2) the hospital physical examination clinic group had a smaller sample size; (3) school-aged children with ASD are often sent to special schools; and (4) clinical symptoms in infants and toddlers might not be typical, and reluctance among parents in the community to acknowledge them could have led to data bias.

Both domestic and international studies [15, 56, 62] have shown a higher ASD prevalence in urban areas than in rural areas, possibly influenced by various environmental pressures or factors [63], such as prenatal exposure to environmental stressors [64] and lower parental education levels [65]. However, the present study indicated that there was no statistically significant difference in ASD prevalence between urban and rural areas. This could be due to the limited number of included studies and small sample sizes, as well as improvements in rural health care and public awareness of ASD in China.

The studies included in this article exhibited a high degree of heterogeneity, which is often challenging to avoid in epidemiological research [66]. To minimize this heterogeneity, we conducted subgroup analyses based on different sampling sources, sampling selection methods, publication years, age, diagnostic criteria, and quality scores. However, due to the limitations of the study designs, subgroup analysis was not performed on screening tools. The 21 studies included in this meta-analysis covered 13 provinces/cities/autonomous regions in China over the past six years, which may not provide comprehensive geographic representation. The sample sizes and representativeness of the regions were limited. Most of the studies did not account for socioeconomic or environmental variables or other relevant factors that could influence the prevalence of autism, thus restricting our ability to study their impact.

## Conclusion

In summary, the prevalence of ASD in China from 2017 to 2023 was estimated to be 7/1000 children, with a male-to-female ratio of 5:1. The overall prevalence remained significantly lower than that reported in foreign countries. The results of this meta-analysis were influenced by limitations in the quantity and representativeness of the studies, as well as potential variations in screening tools and diagnostic criteria between domestic and foreign studies and the comparatively lower level of awareness of ASD in the Chinese population. It is recommended to conduct a nationally representative survey and monitoring of early childhood ASD prevalence to accurately assess its current status and trends. This will provide a more solid foundation for evaluating community service needs and for the establishment and improvement of service systems.

### Electronic supplementary material

Below is the link to the electronic supplementary material.

Supplementary Material 1

Supplementary Material 2

## Data Availability

Data of the studies analysed are already available in publications.

## Abbreviations

ASD: Autism spectrum disorder
AD: autistic disorder
PDD-NOS: pervasive developmental disorder not otherwise specified
ADDM: the Autism and Developmental Disorders Monitoring Network
CBM: China Biology Medicine database
CSTJ: China Science and Technology Journal Database
CNKI: China National Knowledge Infrastructure
AHRQ: the Agency for Healthcare Research and Quality
OR: odds ratios
SMD: standardized mean differences
CI: confidence intervals
C: community-based
K/S: kindergarten/School
H: hospital physcial examination clinic
SCS: Stratified cluster sampling
RCS: Random cluster sampling
RS: Random sampling
RSS: Random stratified sampling
CS: Cluster sampling
NA: Not available
ABC: Autism Behavior Checklist
CABS: Clancy Autism Behavior Scale
CARS: Childhood Autism Rating Scale
CHAT: Checklist for Autism in Toddlers
M-CHAT: Modified Checklist for Autism in Toddlers
CAST: Children Autism Spectrum Test
ASSQ: high function Autism Spectrum screening questionnaire
SCQ: Social Communication Questionnaire
SPT: symbloicplay tes
GDS: Gesell Developmental Schedules
ADOS-2: autism diagnostic observation schedule, 2nd edition
ADI-R: Autism Diagnostic Interview-Revised
ASRS: Social Behavior and Communication Skills Screening Questionnair
WlSC-R: Wechsler Intelligence Scale for Children
SRS: Social Responsiveness Scale
WPPSI: Wechsler Preschool and Primary Scale of Intelligence
WSCMBD: warning sign for children mental and behavioral development Screening Questionnaire
CCMD-3: Chinese Classification of Mental Disorders, 3rd edition
DSM-III-R: Diagnostic and Statistical Manual of Mental Disorders, 3rd edition, revised
DSM-IV: Diagnostic and Statistical Manual of Mental Disorders, 4th edition
DSM-V: Diagnostic and Statistical Manual of Mental Disorders, 5th edition
ICD-10: International Classification of Diseases, 10th revision
